# Lipopolysaccharide Biosynthesis Genes of *Yersinia pseudotuberculosis* Promote Resistance to Antimicrobial Chemokines

**DOI:** 10.1371/journal.pone.0157092

**Published:** 2016-06-08

**Authors:** David L. Erickson, Cynthia S. Lew, Brittany Kartchner, Nathan T. Porter, S. Wade McDaniel, Nathan M. Jones, Sara Mason, Erin Wu, Eric Wilson

**Affiliations:** Department of Microbiology and Molecular Biology, 4007 LSB, Brigham Young University, Provo, UT 84602, United States of America; University of Helsinki, FINLAND

## Abstract

Antimicrobial chemokines (AMCs) are a recently described family of host defense peptides that play an important role in protecting a wide variety of organisms from bacterial infection. Very little is known about the bacterial targets of AMCs or factors that influence bacterial susceptibility to AMCs. In an effort to understand how bacterial pathogens resist killing by AMCs, we screened *Yersinia pseudotuberculosis* transposon mutants for those with increased binding to the AMCs CCL28 and CCL25. Mutants exhibiting increased binding to AMCs were subjected to AMC killing assays, which revealed their increased sensitivity to chemokine-mediated cell death. The majority of the mutants exhibiting increased binding to AMCs contained transposon insertions in genes related to lipopolysaccharide biosynthesis. A particularly strong effect on susceptibility to AMC mediated killing was observed by disruption of the *hldD/waaF/waaC* operon, necessary for ADP-L-glycero-D-manno-heptose synthesis and a complete lipopolysaccharide core oligosaccharide. Periodate oxidation of surface carbohydrates also enhanced AMC binding, whereas enzymatic removal of surface proteins significantly reduced binding. These results suggest that the structure of *Y*. *pseudotuberculosis* LPS greatly affects the antimicrobial activity of AMCs by shielding a protein ligand on the bacterial cell surface.

## Introduction

Recently a significant number of chemokines and chemokine-derived peptides have been shown to possess direct antimicrobial activity, placing them within the broader family of host-defense peptides (reviewed in reference [1]). Host defense peptides, including alpha and beta defensins and cathelicidins, are critical for effective immune protection [2]. While antimicrobial chemokines (AMCs) have been shown to kill a variety of pathogens including viruses, bacteria, fungi and parasites, their mechanisms of direct antimicrobial activity are not well understood. Based on the widespread distribution of chemokines in skin and throughout the respiratory, genitourinary and digestive tracts [3–7], AMCs may represent a previously unappreciated component of the protective barrier at these sites. The localization of antimicrobial chemokines at mucosal portals of entry such as the digestive tract may help regulate infection by pathogenic bacteria.

The chemokines CCL25 and CCL28 both exhibit antimicrobial properties and together their expression profile covers a broad range of mucosal tissues. CCL28 (also known as mucosal epithelial chemokine or MEC) is expressed at high levels in a variety of mucosal tissues, including salivary glands, lactating mammary glands, and the large intestine, with protein levels ranging from 65–232 nM in human parotid saliva [7–10]. The closely related chemokine CCL25 is abundantly expressed in the small intestine [6, 11]. In addition to location-specific differences, chemokine expression may also be influenced by the presence of specific bacterial species [12]. These two chemokines effectively kill a variety of pathogens *in vitro* including Gram-negative and Gram-positive bacteria as well as fungi and protozoa, including both human and veterinary pathogens [7, 13–16].

AMCs and defensins share a conserved motif termed the γ-core, comprising an anti-parallel β-sheet with positive charges distributed at the poles of the motif [17]. It is thought that through their net positive charge and amphipathic structure, defensins selectively target bacteria via attraction to negatively charged phospholipids in the inner and outer leaflets of the membrane, and phosphate groups in lipopolysaccharide (LPS) or teichoic acids. According to this model, electrostatic attraction causes adsorption of peptides onto the bacterial membrane followed by aggregation, integration into the lipid bilayer, and eventual formation of pores, ion channels and cell rupture [18]. Consistent with this model, the antimicrobial activity of CCL28 requires positively charged amino acids in the C-terminus of the protein [13]. AMCs may also cause death by inhibiting cell wall biosynthesis and/or binding to cytosolic targets such as DNA [19, 20]. It is not known how AMCs such as CCL28 and CCL25 recognize bacteria or how bacteria defend themselves against AMCs.

The specific molecular interactions that mediate effective binding to the surface of bacteria and the precise mode of action of AMCs are unknown. While some alpha-helical antimicrobial peptides have recently been shown to target outer membrane lipoproteins such as Lpp in *Enterobacteriaciae* [21], it is not fully understood how other host defense peptides recognize bacterial cells. A clearer understanding of these factors is essential to appreciating how bacteria evade killing by AMCs, and could inform efforts to design novel antimicrobial therapies based on these peptides.

In this study, we sought to identify genes contributing to bacterial resistance to the mucosal AMCs CCL25 and CCL28. Using *Yersinia pseudotuberculosis* as a model, we performed a genetic screen to identify genes that influence bacterial resistance to AMC binding. *Y*. *pseudotuberculosis* is a food- and water-borne pathogen that infects the gastrointestinal epithelium causing an enteric disease characterized by an acute inflammatory response, fever, diarrhea, and abdominal pain. Disease is usually self-limiting but occasionally sepsis, invasion of internal organs, or post-infection autoimmune sequelae such as reactive arthritis occur [22]. Ultimately, control of *Y*. *pseudotuberculosis* infection depends on proper recruitment and activation of phagocytic cells of the innate immune system that kill bacteria through a variety of mechanisms [23, 24]. The abundance of homeostatically produced antimicrobial peptides and chemokines at mucosal surfaces likely serves as a first line of defense against invading pathogens prior to the recruitment of phagocytes. Intestinal bacteria such as *Y*. *pseudotuberculosis* likely encounter AMCs at several points during infection as they traverse the oral cavity and the intestinal mucosa.

**Fig 1:**
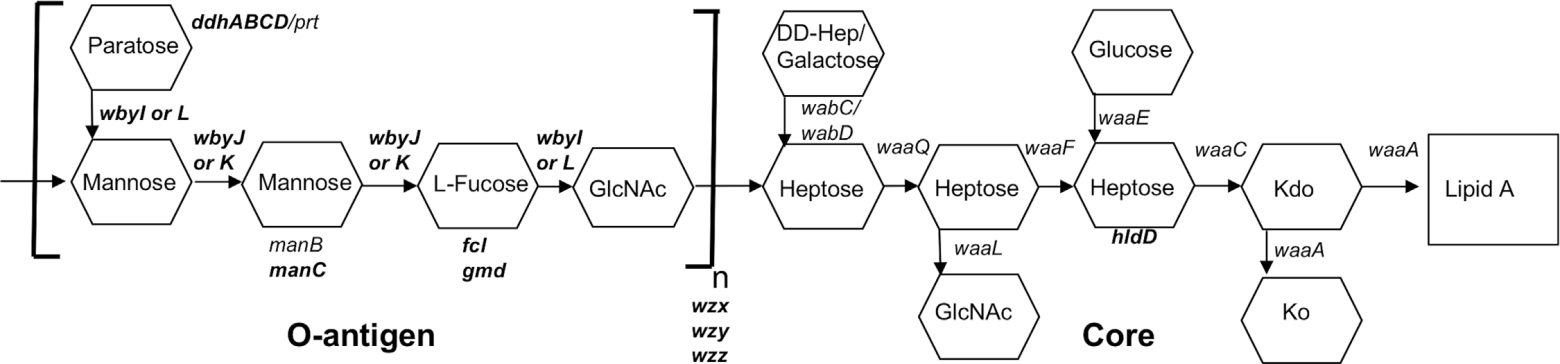
Schematic structure of the *Y*. *pseudotuberculosis* serotype O:1b LPS core and O-antigen. The putative identities of the genes required for LPS synthesis are derived from reference [28]. The O-antigen is predicted to be present in bacteria grown *in vitro* at 21°C but not at 37°C. Those genes highlighted in bold represent genes identified in this study with transposon insertions that result in increased AMC binding.

In this study we sought to investigate AMC resistance factors that would be relevant when bacteria are transmitted by food or water, involving ingestion of *Y*. *pseudotuberculosis* growing at ambient temperatures. *Y*. *pseudotuberculosis* regulates many phenotypes according to temperature. Notably, the LPS O-antigen is maximally produced at temperatures below 30°C [25] and some modifications to the lipid A and core oligosaccharide are also temperature-dependent [26, 27]. We identified several *Y*. *pseudotuberculosis* mutants that exhibited increased binding by CCL25 and CCL28, the vast majority of which were affected in some aspect of LPS synthesis. The predicted core and O-antigen structure of the serotype O:1b IP32953 strain is depicted in Fig 1. Additionally, these high AMC binding mutants were found to be more susceptible to AMC killing than wild type bacteria. Genetic analysis of these mutants demonstrates the importance of the *hldD-waaC* operon and other LPS biosynthesis genes in AMC resistance by *Y*. *pseudotuberculosis*.

## Materials and Methods

### Bacterial strains and culture conditions

A pYV plasmid cured derivative of *Y*. *pseudotuberculosis* IP 32953, a clinical isolate [29], was routinely grown in tryptic soy broth with shaking or on tryptic soy agar or Congo-Red agar (1% heart infusion broth, 0.2% galactose, and 0.01% Congo-Red dye); ampicillin (100 μg/mL) or kanamycin (30 μg/mL) was added when necessary. *Y*. *pseudotuberculosis* was routinely grown at 21°C unless otherwise specified. *E*. *coli* SM10 carrying pRL27 [30] was used as the transposon donor in mutagenesis and *E*. *coli* DH5α was used as a cloning host.

### Transposon mutagenesis and primary screens for high AMC binding mutants

Random transposon mutagenesis in *Y*. *pseudotuberculosis* was performed by biparental mating with *E*. *coli* SM10 harboring pRL27, which contains a Tn5 element encoding kanamycin resistance [30]. Mixtures of log-phase cultures of donor (100 μl) and recipient (300 μl) strains were resuspended in 25 μl fresh broth and incubated overnight at 28°C (to maximize recipient growth) on LB agar with no selection. The mating mixtures were recovered and serial dilutions were plated onto *Yersinia* selective agar containing kanamycin. Approximately 50,000 transconjugants were derived from four separate mating experiments and stored at -80°C in TSA + 15% glycerol. Assuming random insertion of the transposon, this number of mutants would be sufficient for >10X coverage of the *Y*. *pseudotuberculosis* genome. Approximately 15,000 random mutants from this library were grown on Congo-Red agar containing kanamycin at 21°C for 48 h, and colonies that were noticeably darker or lighter than the wild type bacteria were selected for further study. High binding mutants were also selected by subculturing samples of the transposon mutant library for 1 h at 21°C followed by washing in phosphate buffered saline (PBS). The washed bacteria were then incubated with 2.5 nM recombinant CCL28 prepared as described [13] in PBS on ice. The CCL28-coated bacteria were then washed three times in PBS and incubated with biotinylated anti-CCL28 antibody (R&D Systems) for 1 h and then washed in PBS to remove unbound antibody. The bacteria-antibody conjugates were then incubated with streptavidin-conjugated magnetic beads (Miltenyi Biotec) for 30 min and again washed with PBS. Mutants with increased affinity for chemokine were then enriched by passage over a MACS magnetic column (Miltenyi Biotec) and recovered by plating on TSA plates containing kanamycin.

### Arbitrary PCR to identify transposon insertion sites

The locations of transposon insertions in the selected mutants were determined by arbitrary PCR essentially as described by Lauro et al. [31]. For the first reaction, Taq polymerase and primers Arb1 (5’-GGC CAC GCG TCG ACT AGT ACN NNN NNN NNN GAT AT-3’) and intdx (5'—GAG TCG ACC TGC AGG CAT GC—3') or intsx (5'—CGC ACT GAG AAG CCC TTA GAG C—3') targeting either end of the transposon were mixed with bacterial DNA and 5 PCR cycles of 95°C for 30 s, 30°C for 30 s, 72°C for 2 min were performed. This was followed by 25 cycles of 95°C for 30 s, 45°C for 30 s, and 72°C for 2 min. The products of this first reaction were used as templates in the second PCR reaction with primers Arb2 (5'- GGC CAC GCG TCG ACT AGT AC -3’) and nested primers extdx (5'—CCA GAA AGT GAG GGA GCC A—3') or extsx (5'—GAC AAC AAG CCA GGG ATG—3') (30 cycles of 95°C for 30 s, 55°C for 30 s, 72°C for 2 min). The products of the second reaction were prepared for sequencing by digestion with exonuclease I and Antarctic phosphatase to remove free nucleotides and primers. The cleaned PCR products were then sequenced using additional transposon nested primers in BigDye 3.1 (Life Technologies) reactions and capillary electrophoresis at the BYU DNA Sequencing Center. The sequences were then aligned with the *Y*. *pseudotuberculosis* IP32953 genome using Geneious software package (Biomatters) to identify the location and orientation of transposon insertions.

### Plasmid construction

Primers were designed to amplify *hldD*, *hldD/waaF*, and *hldD/waaF/waaC*, including a region of 310 base pairs upstream of *hldD*. The forward primer was the same for each construct (5’-ACG AGT CGT GTC GAG TTG TTG TTC-3’). The following reverse primers, specific for each construct were used: 5’- CAT GGC CTA ACG GCA TTG GAA TTG-3’ for *hldD*, 5’- CGT CAG CGC GGG TAA GGT ATG TAA A-3’ for *hldD/waaF*, and 5’- AGC ACC ACA TTG AGC CGT GGT TAT-3’ for *hldD/waaF/waaC*. PCR products were cloned using the CloneJET cloning kit (Fermentas) and transformed into DH5α cells. Plasmids were isolated from ampicillin resistant clones using the QIAprep Spin Miniprep Kit (QIAGEN) and then cycle sequenced to confirm amplification of the proper gene and also to ensure that the inserts were in the appropriate *hldD/waaF/waaC* orientation relative to the *lac* promoter sequence. Once this was confirmed, complementation plasmids were transformed into the *hldD*::Tn5 mutant strain background via electroporation.

### AMC binding assays

*Y*. *pseudotuberculosis* strains were grown at 21°C (unless otherwise stated) prior to exposure to AMCs in order to simulate the conditions of food or water borne illness. Bacteria grown to mid-logarithmic phase were diluted to 10^6^−10^7^ cfu/ml in filtered PBS supplemented with 0.5 mg/ml bovine serum albumin (BSA). Chemokines (human CCL25 or CCL28, Peprotech) were then added to a final concentration of 250 nM and incubated on ice for 30 min. After three washes in PBS, biotin- conjugated anti-chemokine antibody (R&D Systems) was added and incubated on ice for 30 min and then washed in PBS. Finally, fluorescent streptavidin conjugates (BD Biosciences) were added, incubated for 30 min and washed. Fluorescence was measured using a BD FACSCanto II flow cytometer and analyzed using FACSDiva software (BD Biosciences).

To determine the impact of surface carbohydrates and proteins on AMC binding, bacterial cultures were grown as described above. For oxidation of carbohydrates, the bacteria were then incubated in PBS containing varying concentrations (0–80 mM) sodium periodate (Sigma-Aldrich) for 60 min, followed by neutralization with ethylene glycol, and washed (5X) in PBS supplemented with BSA. For removal of surface proteins, bacteria were incubated in PBS containing 0.1 mg/ml proteinase K (Sigma-Aldrich) for 60 min at 37°C, followed by neutralization with excess BSA, and washed in PBS with BSA. Treated cells were then subjected to AMC binding and detection by flow cytometry.

### Antimicrobial peptide killing assays

Bacteria were grown as described above for AMC binding assays and diluted into filtered 0.1μM potassium phosphate buffer (PPB). After addition of AMCs (250 nM CCL28 diluted in 0.01mg/ml BSA) or BSA (0.01 mg/ml), bacteria were incubated at 21 for 5 h. At the indicated time points, 20 μl of bacteria were removed and put on ice in PBS containing freshly diluted Polybead ® Polystyrene 15 μm Microsphere counting beads diluted 1:62500 (Polysciences Inc). Propidium iodide (PI) (Invitrogen) was added just before reading on the flow cytometer. The number of beads remains constant and the beads can be distinguished from the bacteria on the flow cytometer, which allows the number of viable bacteria to be determined by subtracting PI positive bacteria from the total number of bacteria counted per 30000 beads. Percent survival was calculated by dividing viable bacteria of the sample by viable bacteria in the BSA treated control. Polymyxin B susceptibility assays were carried out in the same manner, except that bacteria were exposed to a range of polymyxin B (Sigma-Aldrich) concentrations (0–20 μM) for 5 h, and then bacterial survival relative to BSA-treated controls were determined by flow cytometry. The statistical significance of specific comparisons of interest was assessed via two-way ANOVA with Dunnett’s correction using GraphPad Prism software (www.graphpad.com).

### LPS isolation and analysis

Bacteria from 1 ml overnight cultures were pelleted and resuspended in 100 μl Tris/Tricine SDS-PAGE sample buffer. Samples were boiled for 10 min, treated with proteinase K and incubated for 1–2 h at 60°C. After another 10 min boiling step, samples were separated by SDS- PAGE using 10–20% gradient gels (Bio-Rad). LPS was stained using the Pro-Q Emerald 300 LPS Gel Stain Kit (Invitrogen) according to the manufacturer’s directions. Gels were then visualized using a UV transilluminator.

## Results

### Identification of *Y*. *pseudotuberculosis* genes affecting AMC binding

Many of the ~27 chemokines that possess antimicrobial activity could impact the ability of *Y*. *pseudotuberculosis* to survive or invade at mucosal surfaces. In this study we focused on the mucosally expressed AMCs CCL25 and CCL28. In order to identify bacterial genes that impact AMC resistance, pools of random transposon mutants were put through two different selection strategies to identify mutants with altered binding to AMCs. In the first approach, we postulated that AMC binding would be influenced by changes in cell envelope structures, which are known to affect adsorption of Congo-Red dye [32, 33]. Normally, *Y*. *pseudotuberculosis* strain IP32953 produces light pink colonies on Congo-Red agar when grown at 21°C. From a pool of ~15,000 random transposon mutants, we identified 88 mutants that exhibited obvious differences in colony morphology and/or Congo-Red binding. These mutants were then subjected to a secondary screen (described below). A second, complementary primary screening strategy approach was employed in which subcultures of the mutant library (50,000 transconjugants) were mixed with a limited amount of CCL28 to ensure that those individual bacteria with higher affinity/avidity were preferentially bound with CCL28. These CCL28-coated bacteria were then incubated with anti-CCL28 antibody followed by a secondary antibody conjugated to a magnetic bead. Mutants with increased affinity for chemokine were then enriched by passage over a MACS magnetic column and 120 single colonies were selected.

For secondary screening, the mutants derived from these two initial selection strategies were grown at 21°C, incubated with CCL25 followed by fluorochrome labeled antibodies, and screened by flow cytometry to measure the amount of AMC bound to the surface of each cell. Both of the initial selection methods yielded mutants with increased AMC binding compared to wild type bacteria. Secondary screening resulted in the identification of 27 of 88 (31%) of mutants derived from the Congo-Red screen and 86 of 120 (72%) of mutants selected through MACS column with increased AMC binding than the wild type strain.

Arbitrary PCR was then used to map the transposon insertion sites of the individual mutants selected during the secondary screen. We were unable to map the transposon insertion sites for 21 of the high-binding mutants (possibly due to multiple insertion events). A further 24 mutants from the MACS selection pool were not considered further because sequencing revealed that they represented clones of the same transposon insertion event. In the remaining 68 unique mutants, the majority of the insertions were found in genes predicted to be necessary for LPS synthesis, including both core oligosaccharide and O-antigen production (Table 1). A schematic of the predicted structure and putative genes required for synthesis of the *Y*. *pseudotuberculosis* serotype O:1b LPS molecule is presented in Fig 1. Multiple independent transposon insertions in the same LPS biosynthesis gene were observed for some genes. For instance, we recovered 11 independent insertion mutants for the *manC* gene, all of which gave very similar binding levels to each other. In addition to the identification of novel resistance genes, this selection approach yielded several genes previously identified as important in resistance to other AMPs, including those coding for phosphoglucomutase (*pgm*) and aminoarabinose modification of lipid A (*pmr*), validating the utility of our approach [34, 35].

**Table 1:** Independent Tn5 insertion mutants in LPS-related genes with increased AMC binding phenotypes.

Hits	IP32953 Locus	Gene Name	Predicted Function	CCL25 binding	
				% +^a^	MF^b^
2	YPTB0055	*hldD*	ADP-L-glycero-D-manno-heptose-6-epimerase	67.6	1097
3	YPTB0263	*rfaH*	transcriptional regulation of capsule/LPS	68.7	430
3	YPTB0998	*ddhD*	CDP-6-deoxy-delta-3,4-glucoseen reductase	50.2	226
2	YPTB0999	*ddhA*	glucose-1-phosphate cytidylyltransferase	43.5	327
1	YPTB1000	*ddhB*	CDP-glucose 4,6-dehydratase	53.8	228
2	YPTB1001	*ddhC*	putative CDP-4-keto-6-deoxy-D-glucose-3-dehydratase	45.5	700
1	YPTB1003	*wbyH*	glycosyl transferase	42.2	487
2	YPTB1004	*wzx*	putative O-unit flippase	32.9	545
1	YPTB1005	*wbyI*	glycosyl transferase, putative	38.5	360
1	YPTB1006	*wbyJ*	putative mannosyltransferase	37.3	322
4	YPTB1007	*wzy*	O-unit polymerase-like protein	43.3	270
4	YPTB1008	*wbyK*	putative mannosyltransferase	45.6	254
3	YPTB1009	*gmd*	GDP-D-mannose dehydratase	74.3	406
10	YPTB1010	*fcl*	GDP-fucose synthetase	65.9	428
11	YPTB1011	*manC*	mannose-1-phosphate guanylyltransferase	67.3	511
7	YPTB1012	*wbyL*	probable glycosyltransferase	52.3	371
2	YPTB1014	*wzz*	O-antigen chain length determinant	68.3	233
2	YPTB2324	*pmrK*	dolichyl-phosphate-mannose-protein mannosyltransferase protein	37.6	688
1	YPTB2328	*pmrI*	probable formyl transferase	62.9	344
1	YPTB2923	*pgm*	phosphoglucomutase	37.0	251
*Y*. *pseudotuberculosis* IP32953 wild type strain				0.6	37

a. Percentage of bacteria with detectable chemokine on surface during chemokine binding assays as measured by flow cytometry.

b. Mean fluorescence of bacterial population following incubation with chemokine and fluorescently labeled anti-chemokine antibody.

**Fig 2:**
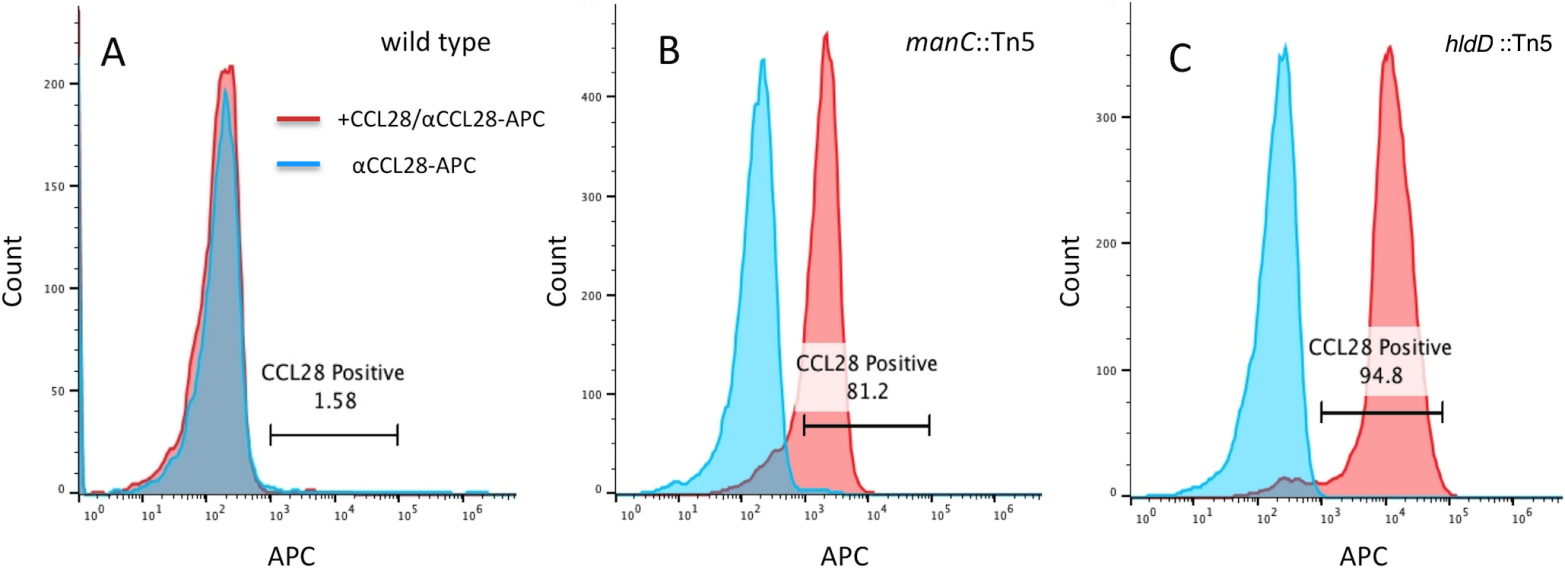
Flow cytometric analysis of CCL28 binding to the surface of wild type and mutant bacteria. In chemokine binding assays bacteria were incubated with CCL28 followed by biotinylated anti-chemokine antibody and APC-streptavidin conjugates. Negative controls included the omission of chemokine followed by the incubation of bacteria with anti-chemokine antibody followed by streptavidin-APC. Results indicate that wild-type *Y*. *pseudotuberculosis* is not readily bound by CCL28 (A). Conversely, transposon mutant insertions in the *manC* (B) or *hldD* (C) genes greatly increase the number of bacteria bound by chemokines.

In this study we performed all primary screening using CCL25. All bacterial mutants were then screened for increased binding to CCL28. This was done in an effort to identify bacterial genes involved in evading the antimicrobial activity of AMCs in general, rather than a single chemokine. Although CCL25 binding was performed in the initial selection, all of the high CCL25-binding mutants also bound readily to CCL28, both in terms of the proportion of the cells stained as well as the fluorescence intensity of the staining (Fig 2 and data not shown). These results suggest considerable overlap in avoidance mechanisms for these two AMCs. Selection of multiple independent insertion mutants in the same gene might be expected to occur because of a particularly strong effect on AMC binding, but we found that this was not necessarily the case. For instance, mutation of *manC* resulted in much more CCL25 and CCL28 binding than wild-type bacteria but the effect of *hldD* was stronger (Fig 2 and Table 1). Therefore, we decided to investigate this gene further.

### The role of the *hldD/waaF/waaC* operon in preventing AMC binding

Both of the transposon mutant screening approaches described above produced mutants with insertions in the *hldD* gene (ADP-L-glycero-D-manno-heptose-6-epimerase). These mutants were among the highest binders of all mutants screened, both in terms of fluorescence intensity and numbers of cells bound (Table 1 and Fig 2), suggesting that the loss of this gene severely inhibited the ability of bacteria to avoid binding by these AMCs. The *hldD* gene, alternatively known as *gmhD*, *rfaD* or *htrM*, is responsible for the synthesis of L, D- heptose, the preferred isomeric form of heptose used for at least the first two heptose residues in the LPS inner core [36]. In *Yersinia pseudotuberculosis*, *hldD* is likely part of a three-gene operon that includes *waaF* and *waaC* (alternatively known as *rfaF* and *rfaC*, respectively). Both are predicted heptosyltransferases, with WaaC likely responsible for transferring the first L, D-heptose residue onto the Kdo residue and WaaF transferring the second [37] (Fig 1). When measured by flow cytometry, the proportion of bacteria that stained positive for CCL28 and CCL25 in these assays was much higher for the *hldD* mutant strain than wild type (Table 1 and Fig 2).

**Fig 3:**
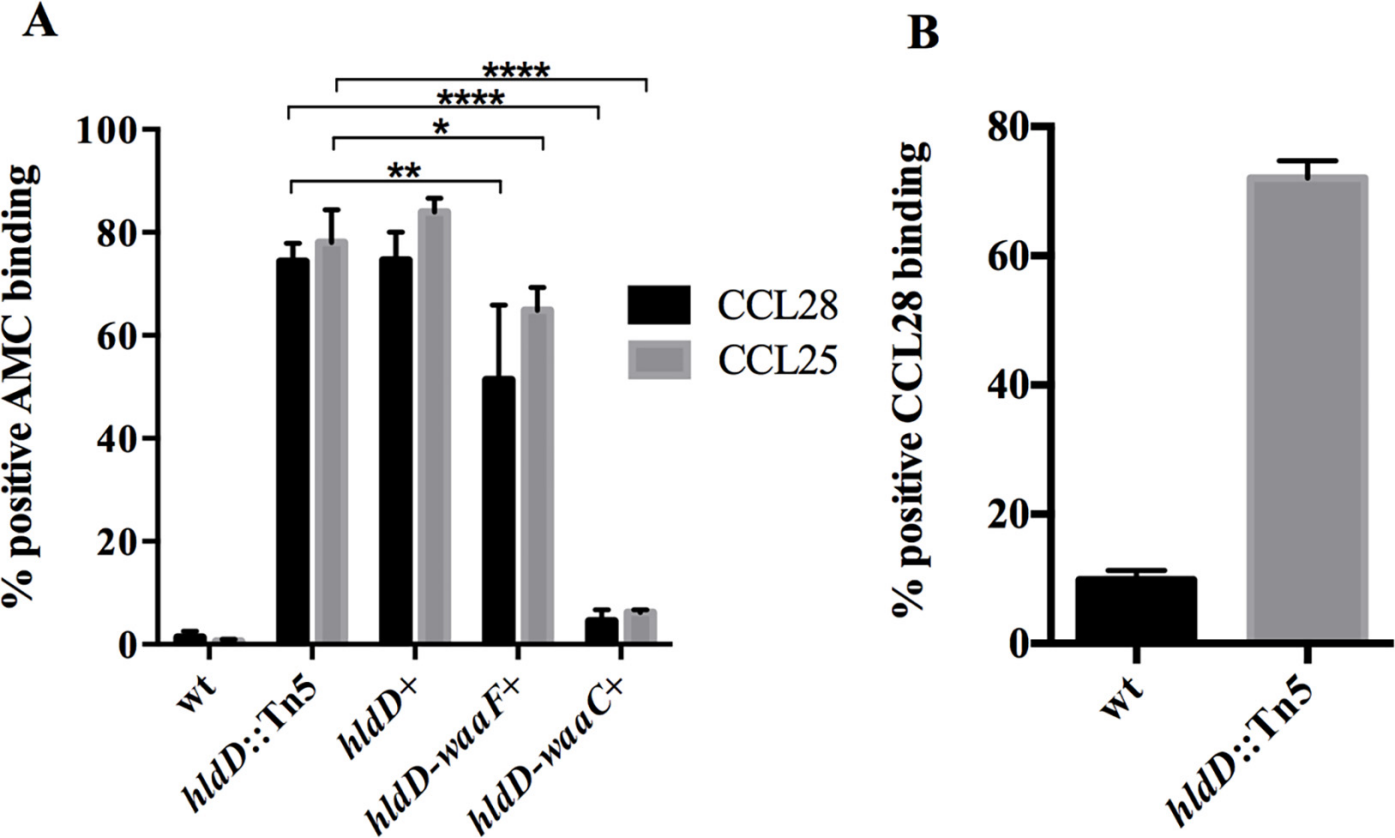
Role of HldD, WaaF, WaaC in AMC binding. (A) A mutation in *hldD* results in significantly greater CCL25 and CCL28 binding (p<0.0001 by Two-way ANOVA), expressed as a percentage of cells that stain positive as measured by flow cytometry. In the *hldD*::Tn5 mutant background, addition of *hldD/waaF* slightly reduced CCL28 (**p<0.01) and CCL25 (*p<0.05) binding, whereas the presence of all three genes (*hldD/waaF/waaC*) completely reduced binding affinity for both CCL25 and CCL28 to wild-type levels (****p<0.0001) when grown at 21°C. (B) Enhanced CCL28 binding due to *hldD* mutation is also evident in bacteria expressing low levels of O-antigen from cultures grown at 37°C.

Transposon insertions in *hldD* could prevent expression of downstream *waaF* and *waaC* genes in the operon. Therefore, we sought to determine the minimum portion of the operon sufficient to complement the wild-type phenotype. This was done through the creation of multi-copy plasmids containing either the *hldD*, *hldD/waaF*, or *hldD/waaF/waaC* genes and transforming these into the *hldD*::Tn5 mutant background. Addition of the *hldD* gene alone had no effect on CCL28 or CCL25 binding to bacteria. Conversely, the addition of the *hldD/waaF* portion of the operon partially restored, and the addition of the entire *hldD/waaF/waaC* operon completely restored the wild type binding patterns for both CCL28 and CCL25 (Fig 3A). Plasmids containing *waaF* and *waaC* alone showed no effect on chemokine binding (data not shown). The inability of the *hldD* mutant strain to produce core oligosaccharide would also preclude the addition of O-antigen. In order to verify the contribution of the core oligosaccharide to resistance to AMC binding, we cultured the wild-type and the *hldD*::Tn5 mutant at 37°C, conditions which are expected to strongly reduce the production of O-antigen (see below). At this growth temperature, the effect of *hldD* mutation on CCL28 binding was also significant, demonstrating that the core oligosaccharide and not just the O-antigen strongly affect binding (Fig 3B).

### The complete *hldD/waaF/waaC* operon is required for resistance to CCL28 mediated killing

Binding of AMCs to a bacterium is a vital first step to mediating microbial death. However, it is also possible that some mutations may cause bacteria to have a strong affinity for AMCs without being killed through this interaction. For instance, some mutants could produce more negatively charged proteins, polysaccharides or lipoproteins on the cell surface that efficiently bind and sequester AMCs, preventing them from reaching their intended targets. Such a scenario would increase AMC binding but not cell death.

**Fig 4:**
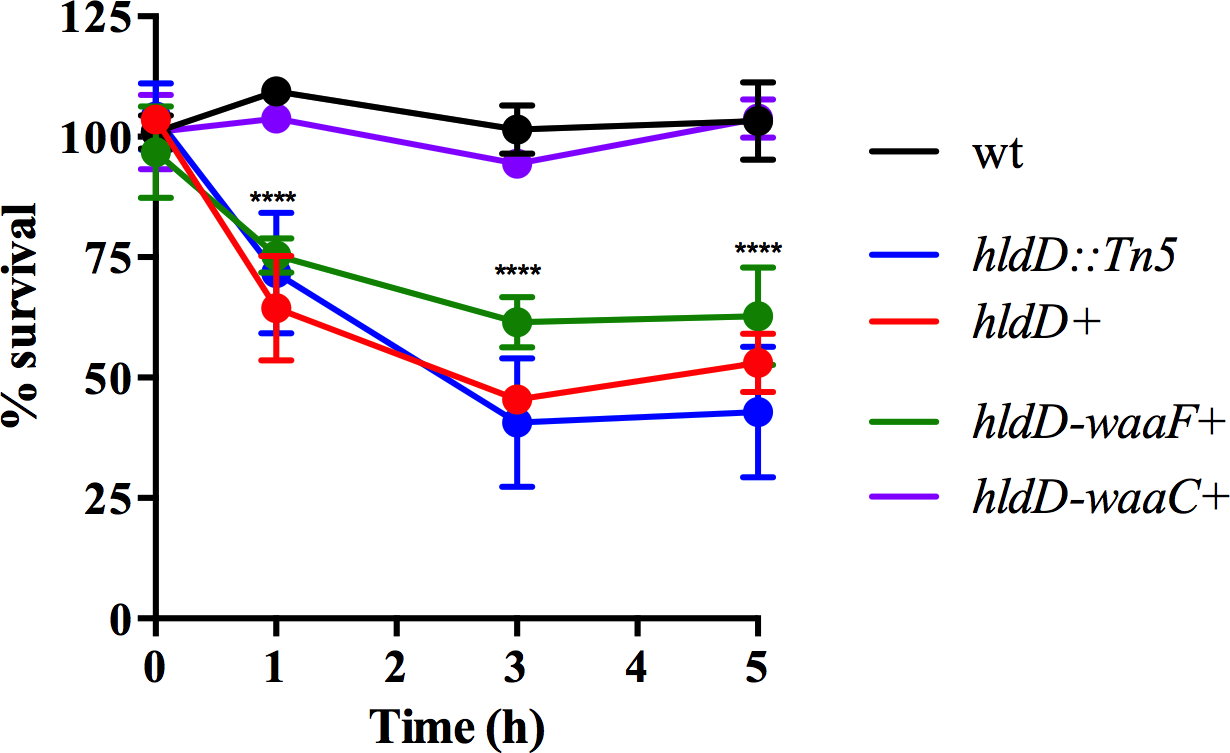
Role of the *hldD/waaF/waaC* operon in survival against CCL28. Relative survival of bacteria (expressed as a percentage of the number of live cells counted in the unexposed control of the same strain) in the presence of CCL28 over a 5 h exposure. Asterisks denote strains that were significantly different from the wild type strain at that time point (Two-way ANOVA (****p < 0.0001).

In an effort to determine whether the increased binding caused by *hldD* disruption results in greater bactericidal activity, we measured bacterial survival in the presence of CCL28 for wild type, *hldD*::Tn5 mutant, and mutant bacteria containing portions of the operon (*hldD*, *hldD/waaF* or *hldD/waaF/waaC*). In these assays, bacteria were treated with CCL28, and their survival relative to BSA-treated controls was measured at 1, 3, and 5 hours of exposure. Fig 4 shows that the *hldD*::Tn5 mutant is significantly more sensitive to CCL28 mediated killing than the wild type strain. A CCL28 concentration that is unable to kill wild type bacteria during a five-hour incubation resulted in approximately 50% mortality in the *hldD*::Tn5 mutant. Addition of the plasmid containing the *hldD* gene alone had no effect on survival of the *hldD*::Tn5 mutant. Bacteria complemented with the *hldD/waaF* portion of the operon exhibited enhanced survival at 3 and 5 hours of treatment compared with the *hldD*::Tn5 mutant strain. Similar to the AMC binding results seen in Fig 3, the full *hldD/waaF/waaC* operon was required to fully restore the *hldD*::Tn5 mutant strain to wild type levels of resistance to AMC mediated cell death.

**Fig 5:**
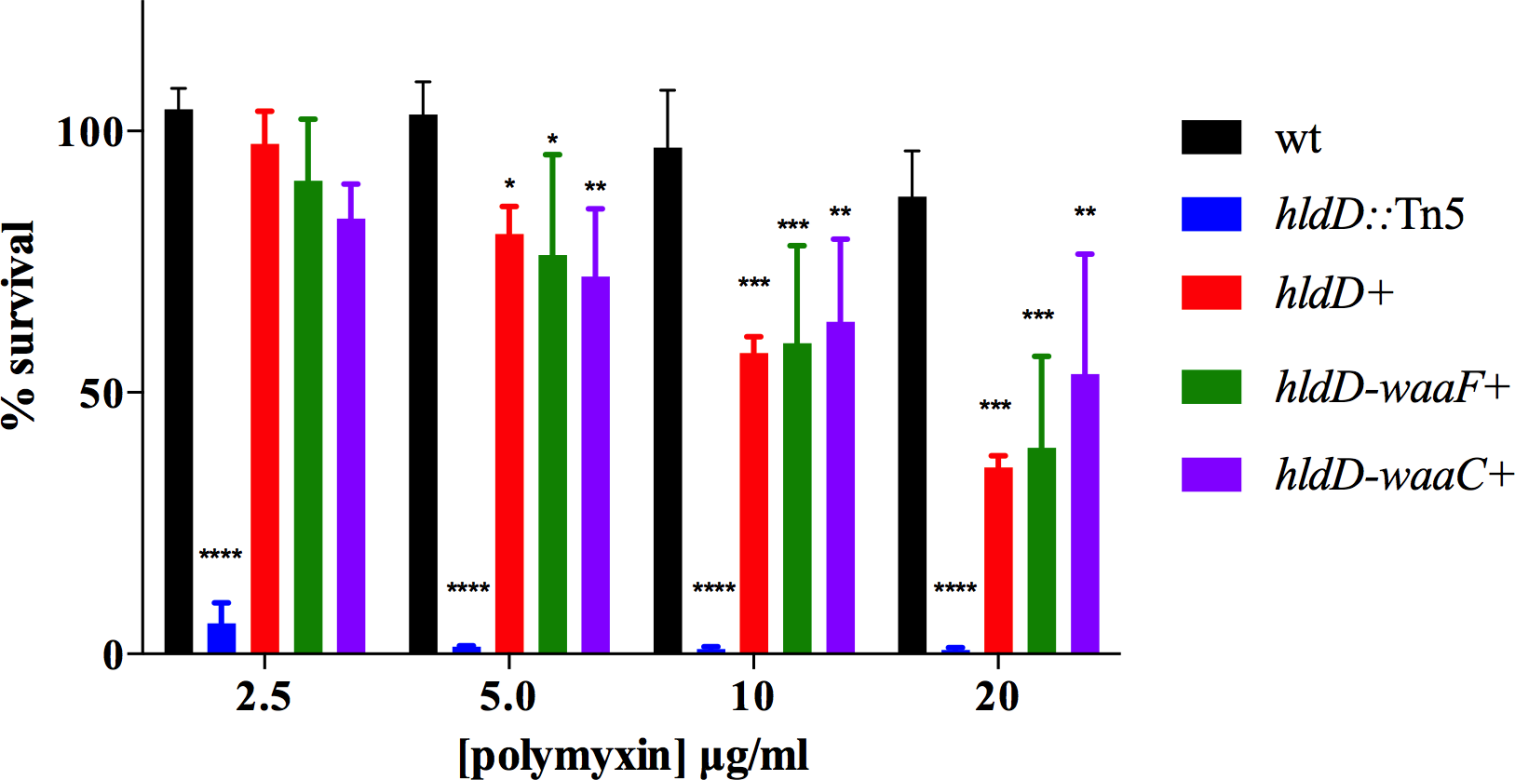
Role of HldD, WaaF, and WaaC in polymyxin resistance. Relative survival of bacteria (expressed as a percentage of the number of live cells counted in the unexposed control of the same strain) in the presence of different concentrations of polymyxin B. Asterisks denote that the result obtained was significantly different from the wild type strain at the given concentration by Two-way ANOVA (****p < 0.0001, ***p < 0.001, **p< 0.01, *p < 0.05).

We next sought to determine if the *hldD/waaF/waaC* operon is important for *Y*. *pseudotuberculosis* resistance antimicrobial peptides other than AMCs. Polymyxin B is a well-characterized antimicrobial protein that disrupts the bacterial membrane and is commonly used as a prototype for assessing susceptibility to host defense peptides. We next tested bacterial survival during a 2 hour exposure to varying concentrations of polymyxin B for the wild type, *hldD*::Tn5 mutant, and mutant bacteria containing portions of the operon (*hldD*, *hldD/waaF* or *hldD/waaF/waaC*) (Fig 5). As expected, the *hldD*::Tn5 mutant was extremely sensitive, with less than 1% survival of bacteria when exposed to 5 μg/ml polymyxin B. The wild type strain was relatively resistant, with no significant reduction in bacterial survival even at 20 μg/ml polymyxin B. Interestingly, the *hldD*, *hldD/waaF* and *hldD/waaF/waaC* plasmids conferred a distinct pattern in the polymyxin B sensitivity tests compared with the CCL28 killing assays. All of the plasmids conferred an intermediate resistance phenotype, yet none conferred full resistance at the highest polymyxin B concentration. In contrast to CCL28-treated bacteria, the presence of *hldD* alone was able to restore some polymyxin resistance compared with the *hldD*::Tn5 mutant. However, the survival rates of the plasmid-containing strains were not significantly different from each other at any of the polymyxin B concentrations. This result implies that in contrast to AMC binding and killing, the production of L, D-heptose (HldD function) is able to provide some protection to the bacteria when treated with polymyxin B, despite the absence of WaaF or WaaC heptosyltransferase activity.

**Fig 6:**
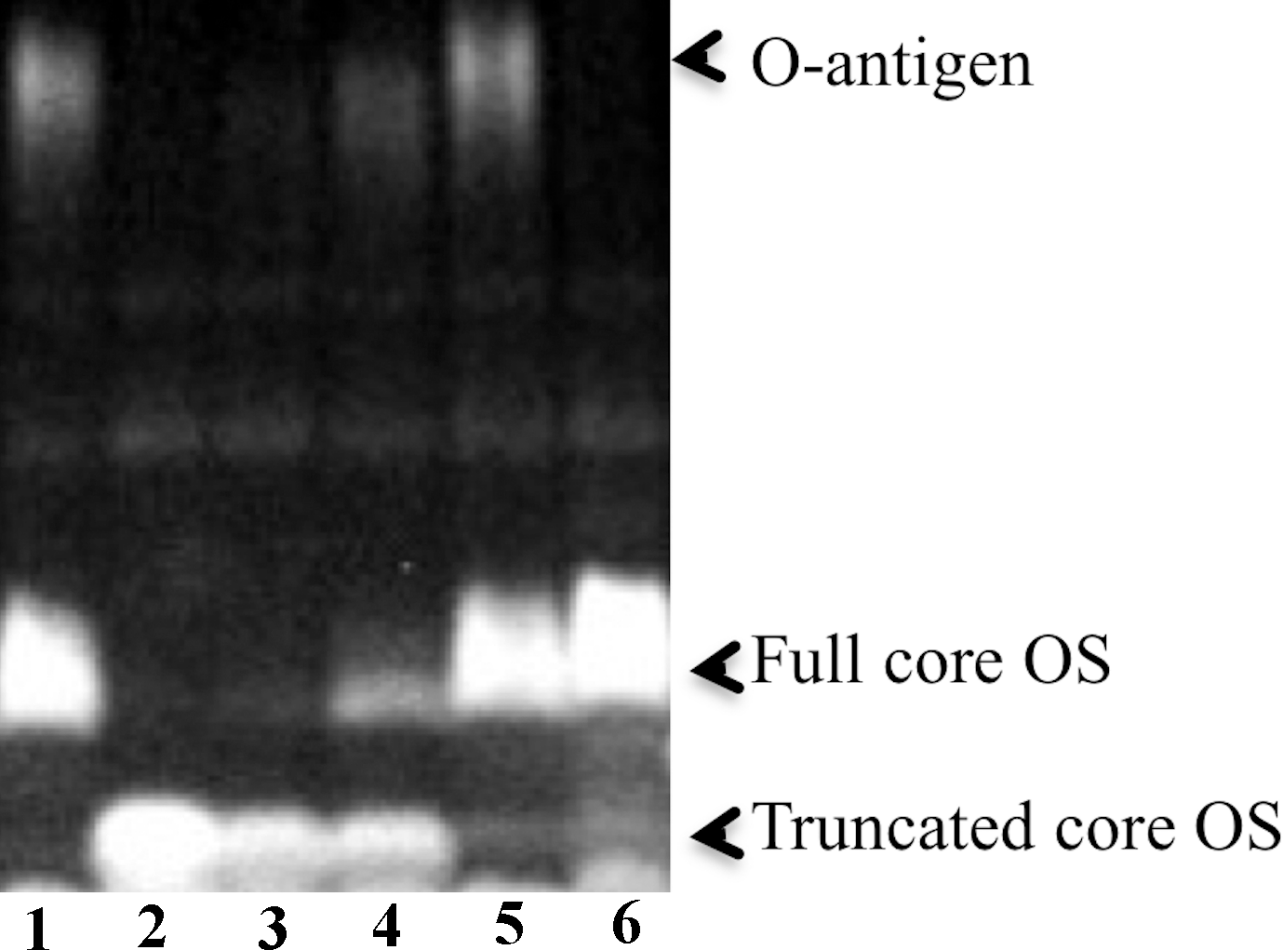
LPS analysis. The *hldD*::Tn5 mutant shows no O- antigen expression and the presence of a truncated core oligosaccharide. Addition of *hldD*, *hldD/waaF*, or *hldD/waaF/waaC* plasmids cause production of full-length core oligosaccharide and O- antigen to increase. As expected, *Y*. *pseudotuberculosis* grown at 37°C does not show O- antigen expression. Sample IDs (from left to right) 1—wild type *Y*. *pseudotuberculosis* IP 32953 grown at 21°C, 2—*hldD*::Tn5 mutant, 3 -*hldD*+, 4 –*hldD/waaF*+, 5—*hldD/waaF/waaC* +, 6—wild type *Y*. *pseudotuberculosis* grown at 37°C. LPS was detected by fluorescent staining of carbohydrates.

In order to better understand the functional importance of these genes in LPS biosynthesis we examined the LPS present in wild type, *hldD*::Tn5 mutant, and mutant bacteria containing portions of the operon (*hldD*, *hldD/waaF* or *hldD/waaF/waaC*). In these experiments LPS was isolated and analyzed on polyacrylamide gels to measure the impact of these genes on the size of LPS components produced by each strain (Fig 6). Core oligosaccharide from the *hldD*::Tn5 mutant migrated faster than that from the wild type strain. This suggests a much smaller core structure, which likely corresponds to lipid A plus KDO or KO. The mutant strains carrying the *hldD* and *hldD/waaF* plasmids showed slight increases in the production of a larger core oligosaccharide. As the presence of larger core species increased in these strains, the abundance of the smaller core structure decreased. The plasmid containing the full *hldD/waaF/waaC* operon resulted in abundant full-sized core oligosaccharide. The wild type strain produced a larger LPS band that we interpreted to be O-antigen. This band was not visible in LPS from *Y*. *pseudotuberculosis* grown at 37°C, which is consistent with previous studies showing O-antigen down-regulation at higher temperatures in other *Y*. *pseudotuberculosis* strains [25]. The *hldD*::Tn5 mutant did not produce O-antigen, but low levels of O-antigen were detected from the mutant bacteria expressing *hldD* or *hldD/waaF*. The strain carrying all three genes (*hldD/waaF/waaC*) produced abundant O-antigen similar to the wild type strain, but small amounts of the truncated core molecule were still detectable. This result suggests that expression of the operon via plasmid greatly enhanced but did not entirely restore production of full length LPS to the entire population of bacteria. This correlates with the absence of full protection against polymyxin B (Fig 5). However, the *hldD/waaF/waaC* plasmid was sufficient to restore protection against killing by CCL28 (Fig 4).

**Fig 7:**
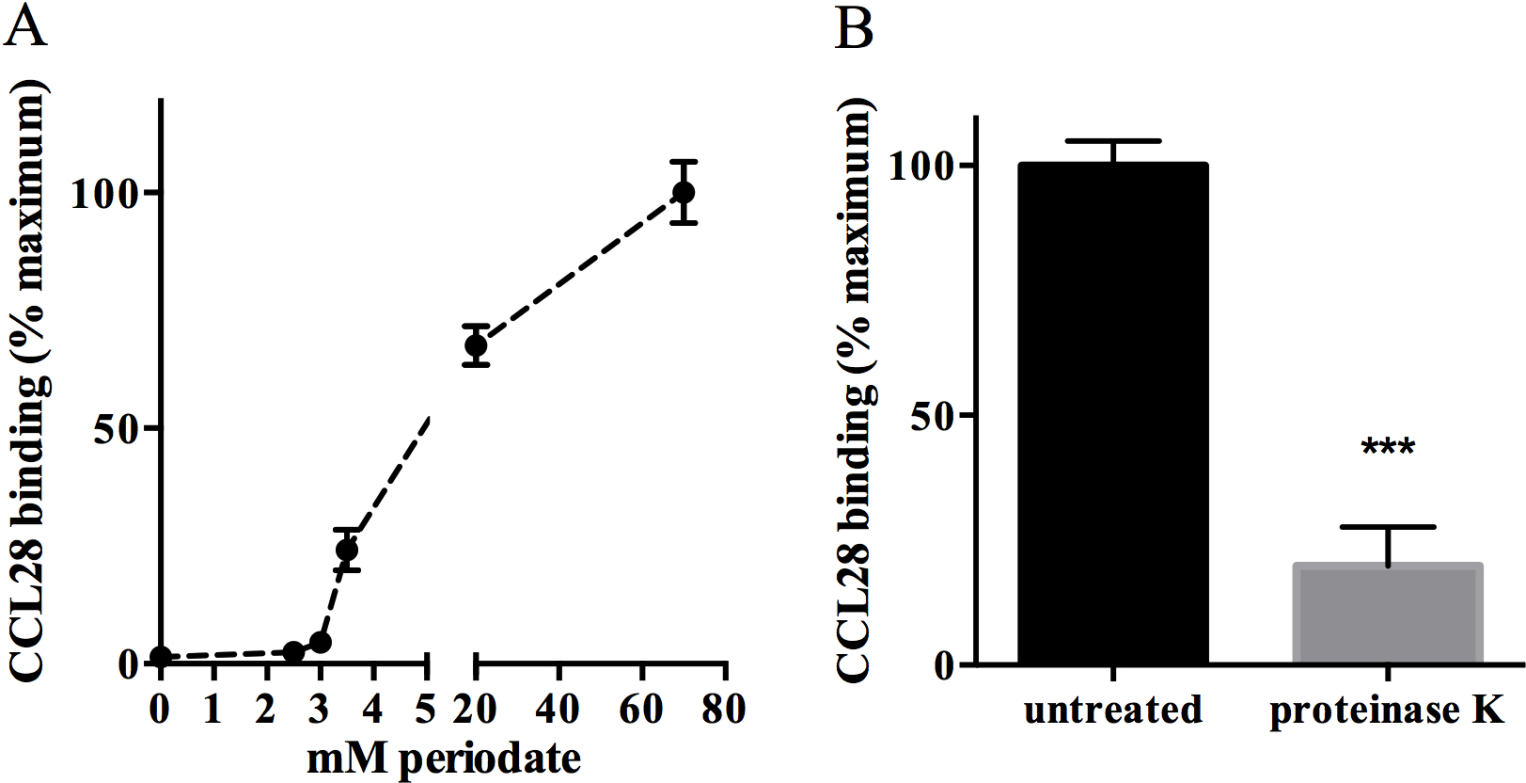
Oxidation of surface carbohydrates increases CCL28 binding. (A) CCL28 binding (fluorescence intensity of the bacterial population after exposure to CCL28) to wild type *Y*. *pseudotuberculosis* IP32953 is enhanced by treatment with sodium periodate in a dose-dependent fashion. The binding levels are expressed as the mean (±SD) relative to the 80 mM samples. (B) Removal of surface proteins from the *hldD*::Tn5 mutant strain by proteinase K treatment decreases CCL28 binding (***p = 0.0001 by unpaired T-test) relative to the untreated samples.

The genetic and LPS analyses described above strongly suggest that the carbohydrate component of LPS is able to repel AMCs from their bacterial target molecules. To confirm the role of surface carbohydrate and further explore the nature of AMC interactions with *Y*. *pseudotuberculosis*, we used a biochemical approach to modify the surface of wild type bacteria and then measured CCL28 binding. In these assays, bacteria were treated with sodium periodate which oxidizes carbohydrates including reducing sugars found in the O-antigen and core oligosaccharide. This greatly enhanced the ability of CCL28 to bind to the surface of *Y*. *pseudotuberculosis* IP32953 (Fig 7A). Treatment with as little as 3.0 mM periodate was sufficient to increase CCL28 binding compared to untreated controls. This suggests that surface carbohydrates may restrict CCL28 binding, and that the bacterial target of CCL28 is not sensitive to oxidation by periodate. To further investigate the nature of the binding target, we treated *hldD*::Tn5 mutant bacteria with proteinase K to remove surface proteins and then measured CCL28 binding. As shown in Fig 7B, treatment with proteinase K diminished CCL28 binding when compared to untreated cells. This suggests that one or more surface proteins are targets of CCL28, or that surface proteins help stabilize target binding.

## Discussion

Several research groups have recently demonstrated the importance of the antimicrobial function of chemokines in defending skin and mucosal tissues against microbial invasion [4, 5, 38, 39]. Our studies are the first to investigate how bacteria avoid the antimicrobial effects of AMCs. A better understanding of the mechanisms used by pathogens to resist these defenses may lead to novel treatments or prevention strategies that enhance the killing efficiency of these innate immune defense proteins. Our results underscore the importance of LPS in the ability of *Y*. *pseudotuberculosis* to resist host antimicrobial defenses. In this study we identified 63 unique mutants with insertions in genes predicted to be involved in some aspect of LPS synthesis or modification that exhibit increased binding to CCL25 and CCL28 when compared to wild type bacteria (Table 1). These results strongly imply that a full length LPS structure is essential for optimum survival in the presence of these AMCs.

Besides the *hldD/waaF/waaC* operon, other *Yersinia* mutations that affect LPS core oligosaccharide or lipid A structure have previously been shown to affect resistance to polymyxin B, beta-defensins, cathelicidins, and cecropins [25, 34, 40–45]. In these reports the effect of specific mutations varied depending on the *Yersinia* species and the antimicrobial peptide being tested. Other research has shown that in several bacteria, the *hldD/waaF/waaC* operon is essential against a range of insults, including bacteriophages, survival in human serum, resistance to opsonization and extracellular neutrophil traps [45–48]. While *Y*. *pestis waaC* has been shown to be required for resistance to polymyxin [45], the role of the *Y*. *pseudotuberculosis hldD/waaF/waaC* operon in resisting host defense peptides has not previously been reported.

We show here that HldD, which provides the L, D-Heptose substrate for the transferases WaaC and WaaF, is crucial for *Y*. *pseudotuberculosis* resistance to CCL25, CCL28, and polymyxin B. We found that all three genes (*hldD/waaF/waaC*) are necessary to confer full resistance to CCL25 and CCL28 binding. We observed slight increases in core LPS structures with a larger molecular weight on Tris-Tricine gels, and marginal binding/killing resistance when *hldD* and *waaF* were expressed together in the absence of *waaC*. This was somewhat surprising since in *E*. *coli* the function of WaaF (heptosyltransferase II) cannot substitute for the function of WaaC (heptosyltransferase I) [49]. This suggests the possibility that the *Y*. *pseudotuberculosis* transferases are less specific than in *E*. *coli* and that WaaF alone may be capable of adding heptose I and heptose II to the Kdo sugar, albeit with much less efficiency.

The pattern of resistance conferred by HldD, WaaF and WaaC was not identical for polymyxin and CCL28. We found that *hldD* alone restores partial resistance to polymyxin B. It is possible that the transposon insertion in *hldD* may not completely abrogate transcription of the downstream genes *waaF* and *waaC*. Although expression of *hldD* alone did not detectably alter the core structure, the sensitivity of our gel assay may not be sufficient to visualize extremely low levels of full-length core oligosaccharide in this strain and more sensitive analysis methods like mass spectrometry would be necessary to confirm this result. Full resistance to polymyxin B mediated killing was not restored even when all three (*hldD/waaF/waaC*) were expressed *in trans*. Gel analysis of LPS from these bacteria showed a small amount of truncated core. Exposing bacteria to polymyxin B may reveal subtler differences among bacterial populations because of the relatively greater potency of polymxyin B compared to CCL25 or CCL28.

In addition to genes essential for core oligosaccharide biosynthesis, we identified transposon mutants with disruptions in genes predicted to be involved in O-polysaccharide synthesis (Table 1), which are clustered between YPTB0998 and YPTB1014 in strain IP32953. This suggests that the absence of O-antigen in *Y*. *pseudotuberculosis* IP32953 (serotype O:1b) has a strong effect on its interaction with AMCs. *Y*. *pseudotuberculosis* produces higher levels of O-antigen at 22°C but less at 37°C (Fig 6, [25]) which may suggest it is not important for virulence in mammals. However, in previously published transposon mutagenesis screens using serotype O3 (YPIII), transposon insertions in *manC*, *fcl*, *wbyJ*, *ddhB*, *ddhC*, *wzx*, and *gmd* reduced the ability of these bacteria to colonize mouse organs following orogastric, intraperitoneal, or intravenous injection [50, 51]. These defects were attributed to the loss of O-antigen, but this was not confirmed by biochemical or structural analysis. Our data suggests that this reduced ability to colonize murine tissues seen previously may be due, at least partly, to increased bacterial sensitivity to AMCs. Thus, if temperature-induced downregulation of the O-antigen genes occurs in vivo, it is possible that it is delayed until after successful establishment within the mucosa or invasion of epithelial tissues [25].

It is possible that this cluster of O-polysaccharide related genes, between YPTB0998 and YPTB1014, impacts the structure of other polysaccharides such as the oligosaccharide core, periplasmic glucans, or enterobacterial common antigen. Although *Y*. *pestis* does not produce O-antigen due to mutations in several genes within the O-antigen gene cluster [52], there has clearly been selective pressure to maintain the other genes in this region, suggesting that these genes contribute in some way to bacterial survival. Perhaps these genes enhance resistance to host defense peptides such as AMCs. The fact that *Y*. *pestis* maintains many of the genes within this cluster without producing O-antigen suggests a separate role for these genes in making other polysaccharides.

In addition to LPS genes, we identified two mutants that contained insertions in genes not known to affect LPS synthesis. These genes are predicted to encode a phosphoribulokinase/uridine kinase (YPTB3726) and an ABC-ribose transport permease subunit (YPTB3806). These genes have not previously been implicated in the evasion of host defense peptides. Two additional mutants had insertions that mapped to intergenic regions. These mutants displayed more moderate increases in their AMC binding compared with the LPS biosynthesis mutants and require further investigation.

In our screens we recovered bacteria with transposon insertions in genes already known to be involved in the ability of *Yersinia* sp. to resist host defense peptides, which confirms the utility of our approach in finding novel resistance genes. For instance, the phosphoglucomutase (*pgm*) gene of *Y*. *pestis* is required for maximum resistance to polymyxin B but not human defensins or LL-37 [34]. Pgm is involved in the biosynthesis of precursors to UDP-sugars, which are incorporated into O-antigen and core oligosaccharides of LPS, as well as other polymers such as cyclic glucans and capsules. The *pmrF* operon is required for aminoarabinose modification of lipid A and confers a high level of polymyxin B resistance, as well as enhancing bacterial survival in phagocytic cells [35, 53]. These results indicate that some of the bacterial characteristics that govern AMC binding also influence the interaction of other antibacterial host defense peptides. Bacterial genes and gene families that control susceptibility to multiple endogenous host defense peptides are particularly exciting as therapeutic targets that could significantly enhance natural innate immunity.

It is still unclear precisely how AMCs target and kill microbial pathogens. However, these studies make clear that full length LPS of *Y*. *pseudotuberculosis* provides protection against cell death. This protection is not due to sequestration of peptide from the binding target, but rather it appears that a full length LPS structure repels, or sterically hinders AMCs from reaching and binding the cell surface. Modification of the LPS through chemical or genetic disruption abrogates this protection. Furthermore, preliminary findings suggest that the target of AMC binding may be a surface protein, as their enzymatic removal from bacterial cells significantly reduces the efficiency of CCL28 binding. This hypothesized protein binding by the AMC is reminiscent of alpha-helical host defense peptides [21] and interferon-γ [54] which bind to lipoproteins on the surface of Gram-negative bacteria to elicit their effects. Further work is necessary to identify the binding target and investigate the consequences of binding that lead to bacterial cell death.
